# Short View of Leukemia Diagnosis and Treatment in Iran

**Published:** 2015-04-01

**Authors:** Mehdi Azad, Ramin Bakhshi Biniaz, Mehdi Goudarzi, Naser Mobarra, Shaban Alizadeh, Hajar Nasiri, Ali Dehghani Fard, Saeid Kaviani, Mohamad Hossein Moghadasi, Mohammad Reza Sarookhani, Mousa Vatanmakan, Mehdi Sahmani

**Affiliations:** 1 Department of Medical laboratory sciences, Faculty of Allied Medicine, Qazvin University of Medical Sciences, Qazvin, Iran; 2 Department of Microbiology, School of Medicine, Shahid Beheshti University of Medical Science, Tehran, Iran; 3 Department of Biochemistry, Metabolic Disorders Research Center, School of Medicine, Golestan University of Medical Sciences, Gorgan, Iran; 4 Department of Hematology, Allied Medical School, Tehran University of Medical Sciences, Tehran, Iran; 5 Hematology-Oncology and Stem cell Transplantation Research Center, Tehran university of Medical Science, Tehran, Iran; 6 Sarem Cell Research Center, Sarem Women’s Hospital, Tehran, Iran; 7 Department of Hematology, School of Medical Sciences, Tarbiat Modares University, Tehran, Iran; 8 Department of Clinical Biochemistry, Cellular and Molecular Research Center, Qazvin University of Medical Sciences, Qazvin, Iran

**Keywords:** Diagnosis, Treatment, Leukemia

## Abstract

**Background: **Early diagnosis and treatment of leukemia patients remains a fundamental aim in clinical oncology, especially in developing country. Present study highlights the basic requirements of these patients in Iran. Better understanding of these issues may lead to improve the healthcare standards toward leukemia diagnosis and treatment.

**Methods: **This descriptive study included 101 specialists in hematology-oncology and pathology serving in oncology centers. The participants were then asked to fill out a standard questionnaire on the issues around diagnosis and treatment of blood malignancies.

**Results: **According to specialists, unfair distribution of facilities across the country, delayed diagnosis of disease, absence of psychological support for patients, and insufficient financial support were the main reasons of inappropriate diagnosis and treatment in leukemia patients.

**Conclusions: **Our results show that making an amendment to health policies by preparing well-equipped medical centers in all provinces, improving the morale of patients through consultation during the process of treatment, and above all, subsiding leukemia patients' financial problems will promote the health standard regarding the leukemia diagnosis and treatment in Iran.

## Introduction

 After cardiovascular diseases, cancers are the second most common cause of mortality in developed countries, and the third in underdeveloped countries. The significance of cancers as a major health problem has been revealed by the increasing incidence over the world by which the economic and psychological status of families can be affected.^   1 ^ Statistically, the rate of cancers in Iran is increasing and it is much higher than standard levels. ^2^^,^^3^  According to the Iranian Ministry of Health (2007), exclusively based on the report of Pathology Center, cancers of skin, breast, stomach, colorectal, liver, hematopoietic system, esophagus, prostate, lung, brain, and the central nervous system (CNS) are the most prevalent malignancies in Iran. ^1^^,^^4^  Leukemia is located in the sixth position of this classification by 5.76% incidence range.^   2 ^ The exact frequency of leukemia is unclear because of the absence of bone marrow biopsy or aspiration availability, especially for children, leading to create falsely low statistics of leukemia.^2^ Therefore, the early diagnosis and control of leukemia are essential to prevent progression of disease. This can be fulfilled by regular screening as a key factor in the ultimate management of diagnostic and therapeutic processes for leukemia patients.^   5 ^

 Patients suffering from leukemia have different predicaments which can be classified as psychological and physical. It is obvious that these two main problems should be resolved in parallel in order to overcome this debilitating disease. ^6^^-^^8^  Psychological needs include supportive and intimate relationship with medical staff,^   9 ^ paying more attention to the mental state of patients for maintaining their spirits and trying to enable them to deal with the new situation.^   10 ^ Physical requirements consist of diagnostic tests and treatment facilities, professional medical teams, diagnosis and treatment of disease and also their treatment costs. Due to the different requirements of leukemia patients, it is essential to scrutinize the weak points of healthcare system regarding leukemia diagnosis and therapeutic standards.^11^ It is essential to say that for such patients, preparing the basic requirements is more important than advanced needs.^   12 ^ The present study has been designed to evaluate the deficiencies in diagnosis and treatment of leukemia in Iran by emphasis on the psychological and physical demands of leukemia patients according to the viewpoint of local specialists. Clear understanding of system defects in leukemia diagnosis and treatment may lead to take much better policies in order to confront these obstacles. 

## SUBJECTS AND METHODS

 A designed questionnaire, which its validity and reliability was confirmed, has been applied in this study. Content-related validity was confirmed based on the specialist's opinions and the latest editions of hematology text books. Moreover, reliability of the test was considered 0.80 by using Cronbach's alpha, which indicated the high internal consistency coefficient of these questions.

In this descriptive study, 101 hemato-oncologists and pathologists serving in medical centers and universities were selected by random cluster sampling method. A questionnaire was classified into two categories according to the previous studies. In the first category, demographic data including age, sex, province, university and last degree were recorded and in the second part fifteen questions were noted down on paper regarding to leukemia diagnosis and treatment. The questions focused on the following issues:

 Availability of diagnostic-therapeutic standards, scientific progress in diagnosis and treatment procedure, available facilities, medical ethics, defects involved the leukemia diagnosis and treatment, scientific level of diagnostic team, information and financial status of patients in improvement of treatment procedure, the satisfaction of leukemia patients with therapy and their tendency toward going abroad (without considering their economic situation), mental status of patients during treatment process, time span from patient admission to beginning the treatment, accuracy and precision of clinical and laboratory examinations for leukemia diagnosis, reliability of leukemia diagnosis methods in comparison with current methods performed over the world, and, at the end, the most common leukemia diagnosis methods in Iran. It should be noted that all questions had 3 or 5 choices and were designed to compare national standards with global standards. The study analyzed the data with SPSS software by means of  a chi- square test. 

## Results

 In this study, we aimed to evaluate the main challenges in diagnosis and treatment of leukemia patients. The age range of the participants in this study was between 29 and 72 years old (mean: 43.22 years) of whom 62.2% were male and 37. 8% were female. Moreover, hemato-oncologists and pathologists consisted 85.15% and 14.85% of this selected group, respectively.  Information of triple-choice questions and their results are indicated in Table 1. According to the results, almost 61% of participants claimed that leukemia diagnostic procedures do not comply with international standards, 23% mentioned that the leukemia diagnostic procedure is not thoroughly acceptable in Iran and 16% asserted that the standard procedures are not performed at all. with the perspective of specialist regarding leukemia research, approximately the same results were obtained; 69% of the specialists believed that poor condition made early diagnosis and more effective treatment difficult or impossible in Iran.

 Comparison of diagnostic equipment in Iran with developed countries showed considerable results. In fact, only 13% of participants in the second-and third-ranked university agreed with appropriateness of existing standards, whereas 65% disagreed. The details of information about diagnostic equipment and methods are shown in Table 2. Regarding academic standards, the majority of participants had a positive response to the question. According to the participants’ responses, awareness and economic situation of patients were 78% and 81%, respectively. However, according to the poor condition, paying attention to patient’s mental health during treatment course may bring promising results. 

 So that 11% of participants chosen "Yes", 42% “somewhat” and 48% “No”. The most common answers to this question whether the elapsed time from the onset of leukemia to definite diagnosis and treatment initiation is the same as international standard levels were negative. On the other hand, accuracy and validity of the diagnostic results were acceptable compared with developed countries. The other results are shown in Table 1.

**Table 1:** Statements related to triple-choice questions about diagnostic standard levels, awareness and economic situation of leukemia patients

**Option items**	**Yes**		** To some extent**		**No**		**Total**	
	**number**	**%**	**number**	**%**	**number**	**%**	**number**	**%**
**leukemia diagnosis procedures in Iran compared with developed countries**	23	22.8	16	15.8	62	61.4	101	100
**Leukemia research trend in Iran compared with the growing global trend**	14	14	17	17	69	69	100	100
**Comparison of diagnosis and treatment facilities of leukemia in Iran with developed countries**	13	12.9	22	21.8	66	65.3	101	100
**Respect to the medical ethics by leukemia diagnosis and treatment team in Iran**	43	43.4	51	51.5	5	5.1	99	100
**Comparison of academic standard level of leukemia diagnosis and treatment teams in Iran with developed countries**	73	73	24	24	3	3	100	100
**Effect of patients’ awareness about leukemia on the diagnosis and treatment procedure**	79	78.2	20	19.8	2	1.98	101	100
**Effect of economic situation of patients in diagnosis and treatment procedure of leukemia**	81	81	17	17	2	2	100	100
**Treatment costs **	20	20	53	53	27	27	100	100
**Paying attention to patient’s mental health during the treatment procedure**	11	11.1	42	42.4	48	48.5	99	100
**Elapsed time from disease onset to initiation of treatment in Iran vs. developed countries**	7	7	35	35	58	58	100	100
**Validity and reliability of leukemia diagnosis in Iran in comparison with developed countries**	72	72.7	21	21.2	6	6.1	99	100

Table 2 shows the items related to five-choice questions. Satisfaction of leukemia patients and their tendency to go abroad for better diagnosis, treatment, and monitoring are major issues discussed in this step. According to participants, there was a high inclination toward pursuing treatment process abroad (regardless of their financial status). Nearly 68% of participants chose “high” and 15% “very high” option, respectively. Two items are specified in Table 3.

**Table 2:** Items related to the five-choice questions on leukemia patient satisfaction rates

**Answers**	**Option item**	**Leukemia patients’ satisfaction**	**Patient´s interest of going abroad for treatment**
**Very high**	Number	6	15
	%	6.1	15.2
**High**	Number	11	67
	%	11.1	67.7
**Moderate**	Number	42	8
	%	42.4	8.1
**Low**	Number	30	4
	%	30.3	4
**Very low**	Number	10	5
	%	10.1	5.1
**Total **	Number	99	99
	%	100	100

 According to study participants, peripheral blood morphology is the most common method to detect leukemia throughout the country, and then it is followed by bone marrow morphology and clinical findings. Also, the participants mentioned that flow cytometry is another important method for leukemia diagnosis, especially in case of acute leukemia. On the other hand, all participants cited that peripheral blood, BM morphology and clinical findings were easily accessible diagnostic procedures, but the accessibility of flow cytometry, molecular genetics and cytogenetic techniques were confirmed by 37%, 17.8% and 8.9% of participants, respectively. 

 Only 7.9% of the participants confirmed that all above-mentioned diagnostic procedures were available in their facilities. Peripheral blood, BM morphology and clinical findings are available diagnostic methods in our cities, but none of the other methods listed in Table 3 were accessible. Nowadays, flow cytometry is currently the most common method for diagnosis of leukemia. In towns, the samples are frequently sent to larger cities for flow cytometric analysis but only the towns that are near big cities like Tehran have this chance and remote towns have serious problems.

## Discussion

 In this study, we aimed to design and explore a new kind of research which is based on medical specialist views about the present issues in diagnosis and treatment procedures of leukemia patients. Findings revealed that the most important problem is the shortage of equipment and unequal distribution of facilities throughout the country, especially for flow cytometric analysis, molecular studies and cytogenetic assessment. For example, molecular studies are just performed in major cities such as Tehran, Isfahan, Mashhad, Shiraz, Kerman and Tabriz (although not perfectly), whereas small cities lacking adequate facilities must send their samples to larger ones for analysis, which can be a waste of money and time. Moreover, it can prolong the process of diagnosis and delay the treatment, resulting in significant effect on therapeutic outcome in leukemia patients and making diagnosis difficult in Iran. 

 In general, economic problems and sanctions are the most important reasons for current shortage of equipment. Another finding was about the time elapsed from diagnosis of leukemia to initiation of therapy in Iran compared with developed countries. The 35% of specialists who were primarily employed at top-ranked universities in large cities expressed that this period of time is relatively favorable, and 58% (at Second- and third- ranked universities) declared unacceptable. These findings are consistent with findings of facility shortage. 

 Obviously, there is a direct correlation between available facilities and the span of time between patient admission and definite diagnosis. According to our survey, even in major cities, there are only a few well-equipped centers. 

 In this study, only 7.9% of the participants stated that they had full access to all necessary equipment in their centers, viewing diagnostic process as favorable to developed countries. One of the main reasons that reduce the chance of treatment for acute leukemia patients in Iran is maybe the lack of a screening system for punctilious diagnosis. In other words, the average rate of timely diagnosis of acute leukemia in Iran is children and women, causing poor outcomes.

**Table 3:** Items related to five-choice questions on topics of common diagnostic methods and the overall availability of diagnostic methods

** Answers**	**Option item**	**Most common diagnostic methods**	* **Availability of diagnostic methods**
**Peripheral blood morphology**	Number	36	101
	%	36	100
**BM morphology**	Number	30	101
	%	30	100
**Clinical findings**	Number	28	101
	%	28	100
**Flow cytometric analysis**	Number	4	37
	%	4	36.6
**Molecular studies**	Number	1	18
	%	1	17.8
**Cytogenetic**	Number	1	9
	%	1	8.9
**All items**	Number	0	8
	%	0	7.9
**Total participants**	Number	100	101
	%	100	100

* Participants could choose more than one option

Another question that was considered in the questionnaire was about the optimization of leukemia diagnosis and treatment procedures in Iran compared with global state. The 69% of specialists replied in the negative and asserted that the existing trend is unfavorable. The results regarding the question which underscored the availability of research facilities in academia indicated that slow rate of leukemia investigations in Iran is probably associated with economic problems and sanctions which directly affect the quality and quantity of existing research facilities. Another question was regarding the patient’s mental health during the treatment course. Only 11% of specialists replied positive to the question and 48% chose the "No" answer, which seems to be reasonable. Thus, psychologists, social workers and counselors may have a profound impact on the treatment course. Accumulating evidence has demonstrated the dramatic effects of psychological issues on cancer patients^   13 ^ Remmers et al. (2010) considered the emotional support as an urgent requirement for women with breast cancer.^   14 ^ Heydari et al. (2009) stated that constructing social support network for cancer patients via support groups including physicians, nurses and relatives or friends of patients is important.^   15 ^ Interestingly, the most important point in increasing the self-confidence and improving mental state of patients is drawing their attention to spiritual matters. Tatsumura et al. (2002) indicated that cancer patients who rely on spiritual and religious beliefs can better handle the stress of illness during the treatment course.^   16 ^ Additionally, Harandi et al. (2010) concluded that paying more attention to emotional, social and mental health of patients are beneficial for patients.^   11 ^ Taghavi et al. (2011) showed that Cancer Support Community is a source of social support along with other resources^   17 ^ and can be helpful in terms of both emotional and financial support for cancer patients.  Various societies are active in this field in Iran but it seems that their activity is not enough to fill the existing gaps. The study of Harandi et al. (2010) confirmed that one of the basic problems in cancer patients is the lack of associations and institutions to support those requirements.^   11 ^ The charitable activities of these associations cannot satisfy our expectations, so effective government agencies are required to meet the ends and fill the gaps. Ismaili et al. (2010) showed that aid organizations (government, non-government and community-based organizations) can make a major contribution in response to patients' needs.^   18 ^ Financial support provided by these organizations, including health insurance, will be crucial and decisive, especially in the diagnosis and treatment of cancer. Another part of our study focused on the answer to this question whether patients' knowledge about leukemia has effect on timely diagnosis and treatment. Based on the results obtained in this study, 78% of the participants replied positive to the question. Our findings show that patients' knowledge has an important role in the timely diagnosis and treatment of the disease. Therefore, the patient’s knowledge about their treatment course is an important requirement for diagnosis and another aspect of protecting leukemia patients. Jenkins et al. (2001) stated that most patients tend to receive much more information in relation to the disease and its treatment.^   19 ^ Drageset et al. (2010) concluded that information about the disease from medical or other sources is helpful for cancer patients.^   20 ^ Esbebsen et al. (2010) found that lack of information may guide patients to unreliable sources of information about their disease.^   21 ^ Our study also demonstrated that the poor economic condition of leukemia patients along with other problems is one of their major predicaments. According to the Yan and Sellick. (2004) financial issues and mental health counseling are important to deal with the concerns of cancer patients.^   22 ^ We found that one of the fundamental problems in leukemia patients is overwhelming costs they spend for diagnosis and treatment in Iran.

 Present study is the first investigation of this kind in Iran. But the exact correlation between these problems and mortality or remission of patients has suffered most neglect and needs  most detailed scrutiny. Knowing the gaps and deficiencies allows us to develop healthcare strategic plans. It is obvious that resolving current problems and filling the existing gaps will improve health standards in the future. 

## CONCLUSION

 Making an amendment to the policies governing the healthcare system should lead to fair distribution of facilities across the country, acceptable attention to the mental state of patients by therapist and appropriate counseling centers during the treatment process, and ultimately financial support of government and non-government organizations for leukemia patients, especially low-income families, will certainly pave the way to achieve the much better health standards regarding the leukemia diagnosis and treatment in Iran.
